# *GCKR* common functional polymorphisms are associated with metabolic syndrome and its components: a 10-year retrospective cohort study in Iranian adults

**DOI:** 10.1186/s13098-021-00637-4

**Published:** 2021-02-18

**Authors:** Asiyeh Sadat Zahedi, Mahdi Akbarzadeh, Bahareh Sedaghati-Khayat, Atefeh Seyedhamzehzadeh, Maryam S. Daneshpour

**Affiliations:** Cellular and Molecular Endocrine Research Center, Research Institute for Endocrine Sciences, Shahid Beheshti University of Medical Sciences, POBox: 19195-4763, Tehran, Iran

**Keywords:** Metabolic syndrome, Triglyceride, Single nucleotide polymorphisms, GCKR

## Abstract

**Background:**

Previous studies reported that common functional variants (rs780093, rs780094, and rs1260326) in the glucokinase regulator gene (*GCKR*) were associated with metabolic syndrome despite the simultaneous association with the favorable and unfavorable metabolic syndrome components. We decided to evaluate these findings in a cohort study with a large sample size of Iranian adult subjects, to our knowledge for the first time. We investigated the association of the *GCKR* variants with incident MetS in mean follow-up times for nearly 10 years.

**Methods:**

Analysis of this retrospective cohort study was performed among 5666 participants of the Tehran Cardiometabolic Genetics Study (TCGS) at 19–88 years at baseline. Linear and logistic regression analyses were used to investigate the metabolic syndrome (JIS criteria) association and its components with rs780093, rs780094, and rs1260326 in an additive genetic model. Cox regression was carried out to peruse variants’ association with the incidence of metabolic syndrome in the TCGS cohort study.

**Results:**

In the current study, we have consistently replicated the association of the *GCKR* SNPs with higher triglyceride and lower fasting blood sugar levels (p < 0.05) in Iranian adults. The CT genotype of the variants was associated with lower HDL-C levels. The proportional Cox adjusted model regression resulted that TT carriers of rs780094, rs780093, and rs1260326 were associated with 20%, 23%, and 21% excess risk metabolic syndrome incidence, respectively (p < 0.05).

**Conclusions:**

Elevated triglyceride levels had the strongest association with GCKR selected variants among the metabolic syndrome components. Despite the association of these variants with decreased fasting blood sugar levels, T alleles of the variants were associated with metabolic syndrome incidence; so whether individuals are T allele carriers of the common functional variants, they have a risk factor for the future incidence of metabolic syndrome.

## Background

The metabolic syndrome (MetS) represents a combination of metabolic abnormalities that increase the risk of developing Type 2 diabetes mellitus by fivefold, cardiovascular diseases (CVDs) by twofold, and the risk of all-cause mortality by 1.5-fold [1, 2]. These metabolic abnormalities include insulin resistance, elevated blood pressure, dyslipidemia, and central obesity [3]. So, early diagnosis of metabolic syndrome and evaluation of its predisposing factors can be as valuable as the study of diabetes biomarkers [4, 5] and strong biomarkers of cardiovascular disease [6–8] in the prevention and treatment of two disorders. The pathophysiological mechanism that is explicating MetS remains unclear, and maybe more than one. It is noticeable that lipid profile and glucose metabolism change due to central obesity and insulin resistance, and this change leads to the development of MetS [9]. The average worldwide prevalence of metabolic syndrome is 31% [10]. In comparison, according to the International Diabetes Federation (IDF) criteria, the prevalence of this disorder in Iranian adults estimate 37.4% [11] and, based on a meta-analysis of published data during 2000–2016, is higher in some ethnic groups (Bushehr 57.8%) [12]. Therefore, it is crucial to consider the factors that contribute to metabolic syndrome as a health threat. MetS is a multifactorial disease, and besides environmental factors, genetic studies have revealed susceptible loci in associations with MetS [13].

In the liver and the Langerhans islets’ beta-cells, glycolytic enzyme glucokinase (GCK) contributes to glycogen synthesis regulation and gluconeogenesis as a primary glucose sensor [14]. In the first step of glycolysis, GCK is responsible for glucose phosphorylation; thus, GCK carries out a pivotal role in maintaining blood glucose homeostasis [15]. Glucokinase regulatory protein (GKRP) regulates glycolysis by inhibiting GCK enzymatic activity at low glucose concentrations. When glucose concentration rises, the GCK/GKRP complex disconnects so that more GCK are available, leading to increased hepatic glucose utilization [16]. GCK activity rises through overexpression of the glucokinase regulator gene (GCKR) in the liver, leading to decreased glucose and increased triglyceride concentrations [17].

Glucokinase regulator gene (*GCKR*) locates on chromosome 2p23.3-p23.2 and containing 19 exons, encodes GKRP (68 kDa, 625 amino acids) [18, 19]. Genome-wide association studies (GWAS) and multiple candidate gene studies have reported that *GCKR* genetic variation is associated with metabolic parameters such as triglyceride (TG) levels [20–26], fasting blood sugar (FBS) [21–23, 26–28], and insulin [27, 28] or metabolic disorders like type 2 diabetes (T2DM) [27, 29], dyslipidemia (high triglyceride and low high-density lipoprotein cholesterol levels) [23, 30]. Common functional variants, rs780094, rs780093, and rs1260326, are broadest studied genetic variants of the *GCKR* gene [21, 24, 26, 29, 30]. The T allele of mentioned SNPs is associated with FBS concentration reduction, insulin level, lower insulin resistance, and T2DM prevalence. Otherwise, the associations with higher fasting serum triglyceride concentration in several populations were reported [21–24, 26, 29, 30]. This opposite effect on both TG and insulin traits did not affect the association between the T-allele of the variants and MetS in some previous findings [30, 31].

Association between common polymorphisms of the *GCKR* gene and MetS investigated in a few studies among the Iranian population; Most of them performed during childhood. None of which focused on the associations between the *GCKR* variants and MetS components. Earlier in a cross-sectional study, we confirmed the association of rs780094 and rs1260326 with an increased risk of developing metabolic syndrome and its components, published in Persian [32].

In the present study, a retrospective cohort approach, we investigated the association of *GCKR* variants (rs780093, rs780094, rs1260326) with MetS incidence and its components in new cases MetS by utilizing data of the Tehran Cardiometabolic Genetic Study (TCGS).

## Methods

### Study population

Subjects of this retrospective cohort study were selected from TCGS participants [33]. TCGS is an ongoing genetic study that is a part of the Tehran Lipid and Glucose Study (TLGS). This cohort study estimates the prevalence of non-communicable disease risk factors in a sample of Tehran’s (capital of Iran) 13th district residents [34]. The first survey of the TLGS was initiated from 1999 to 2001 on 15,005 individuals aged 3 years, and subjects were genotyped and followed up to identify recently developed diseases every 3 years. In the current study, we excluded individuals with prevalent MetS at baseline. A total of 5666 adults 19–88 aged years who had complete demographic, anthropometric, biochemical, and genotype baseline data were included in the statistical analyses. All subjects’ written consent was obtained, and the Research Institute for Endocrine Sciences (affiliated to Shahid Beheshti University of Medical Sciences, Tehran, Iran) approved this study.

### Measurements

At each survey of TLGS, participants signed a consent form. Information for age, sex, and history of using medication for diabetes, hypertension, smoking, and lipid disorders collected with a standardized questionnaire. Anthropometric measurements were recorded using standard protocols; waist circumference (WC) was measured and recorded to the nearest 0.1 cm using a non-stretch tape meter. Systolic blood pressure (SBP), diastolic blood pressure (DBP) were measured as described previously [34]. A blood sample was drawn after 12–14 h overnight fasting. Samples were centrifuged within 30–45 min of collection, and the sera were used for biochemical measurements. FBS, TG, and high-density lipoprotein cholesterol (HDL-C) were measured by the enzymatic colorimetric method, using commercial kits (Pars Azmoon, Tehran, Iran); also, coefficients of variation (CV) for HDL-C and triglyceride measurements were below 5%.

### Genetic analysis

To select SNPs in *GCKR*, we searched for variants whose function was identified and proven. In previous studies, the function of rs1260326 is described as reducing the inhibitory properties of the GKRP, which leads to increased glucokinase activity in the liver [35, 36]. The rs780093 and rs780094 are in strong linkage disequilibrium with this functional variant in different populations [37]; therefore, we investigated these three variants’ effects on metabolic syndrome in the Iranian population.

Genomic samples were extracted from the buffy coat using the standard Proteinase K, the salting-out method. DNA samples were genotyped with HumanOmniExpress-24-v1-0 bead chips (containing 649,932 SNP loci with an average mean distance of 4 kb) at the deCODE genetics company (Iceland) according to the manufacturer’s specifications (Illumina Inc., San Diego, CA, USA). Quality control procedures were performed by the PLINK program (V 1.07) and R statistic (V 3.2), and the genotyping data of *GCKR* polymorphisms (rs780094, rs1260326, and 780093) were used for present association analysis.

### Study outcomes

Outcomes were the components of MetS as continuous and categorized traits [30], and the prospective outcome was incident MetS. The MetS were defined According to the most recent Joint Interim Statement (JIS) of the International Diabetes Federation Task Force on Epidemiology and Prevention [32]. Individuals were considered with metabolic syndrome if they had at least three of the following metabolic components in at least one survey of TCGS: central obesity (waist circumference ≥ 90 cm for both genders based on the Guidelines of the Iranian National Committee for Obesity [38]); elevated TG (fasting serum TG ≥ 150 mg/dl and drug treatment); reduced HDL-c (fasting serum HDL-c < 40 mg/dl in men and < 50 mg/dl in women and drug treatment); elevated blood pressure (SBP ≥ 130 mmHg or DBP ≥ 85 mmHg or taking hypertension medication); high fasting glucose (FBS ≥ 100 mg/dl or taking diabetes medication).

### Statistical analysis

SPSS software version 21.0 (SPSS Inc. Chicago, IL, USA) was used for most statistical analyses. Only we calculated pairwise linkage disequilibrium (LD) using the "genetics" package in R. The baseline characteristics were presented as means ± standard deviation (SD) for continuous traits or as number (percentage) for categorical traits. Hence, serum triglyceride levels were not normally distributed, so their natural logarithm (Ln) was taken before analysis. *P-*value < 0.05 was considered significant. Chi-square test was used to investigate deviations of observed genotype frequencies from those predicted by the Hardy–Weinberg equation and for determining the frequency of genotypes in metabolic trait groups.

Chi-square test and logistic regression were performed to test the association between the MetS and its components as categorized traits and the three variants. To reduced heterogeneity of differing lengths of follow-up in odds ratios and considered the duration of follow-up of censored observations, we estimated the hazard ratio and 95% confidence interval of the incidence of MetS. Two Cox models were performed; model 1 unadjusted model, and model 2 was adjusted for age (baseline for Mets and without MetS), gender, and smoking. We defined time to event for participants with MetS as the mid-time between the date of the follow-up visit when the Mets were diagnosed for the first time and the most recent follow-up visit before the diagnosis, and for participants without MetS, the time between baseline to the last follow-up. The effects of the *GCKR* polymorphisms on the MetS components as quantitative traits were evaluated first by analyzing variance, ANOVA followed by posthoc Tukey’s test, and then utilizing linear regression models. In examining the association between the SNPs and MetS components (categorized or quantitative) for individuals with MetS, the first occurrence of MetS in the follow-up period was considered the baseline.

## Results

### Population characteristics

The population’s average age was 39.2 years in the baseline, and 57.4% were female.  During the follow-up period of about ten years, two thousand eight hundred eighty-four subjects (50.9%) developed MetS in at least one survey of the present study. Analysis of the distribution of MetS components demonstrated that low-level HDL-C was the most common component of metabolic syndrome, which observes in 69.8% of subjects. Increased levels of fasting glucose had the lowest frequency (22.3%) (Table 1).

**Table 1:** Baseline characteristics of participants

	Total	rs780094			rs780093			rs1260326		
		CC (n = 2543)	CT (n = 4169)	TT (n = 1879)	CC (n = 2509)	CT (n = 4180)	TT (n = 1902)	CC (n = 2478)	CT (n = 4174)	TT (n = 1939)
With MetS	50.9%	28.1%	49%	23%*	28.2%	48%	23.8%*	27.4%	49.0%	23.6%*
Without MetS	49.1%	32.5%	47.1%	20.4%*	31.6%	48.2%	20.2%*	31.4%	47.7%	20.9%*
Male	42.6%	30.9%	47.8%	21.2%	30.8%	47.5%	21.6%	30.3%	48.0%	21.8%
Female	57.4%	29.7%	48.0%	22.2%	29.3%	48.4%	22.3%	28.8%	48.5%	22.7%
Smoking never	83%	30.6%	47.9%	21.5%	30.3%	48.0%	21.7%	29.7%	48.4%	21.9%
Ex-smoker and smoker	17%	28.0%	48.9%	23.1%	28.0%	49.0%	23.0%	27.8%	48.2%	23.9%
Age (years)	39 ± 16	39 ± 17	39 ± 16	40 ± 17	39 ± 17	39 ± 16	40 ± 17	39 ± 17	39 ± 16	39.5 ± 16.6
WC (cm)	85 ± 21 (53.3%)^a^	85 ± 21	85 ± 21	85 ± 22	85 ± 22	85 ± 21	85 ± 22	85 ± 22	85 ± 21	85.4 ± 21.5
HDL-C (mg/dl)	42 ± 14 (69.8%)^b^	43 ± 14	42 ± 14	42 ± 15	43 ± 14	42 ± 14	42 ± 14	43 ± 14	42 ± 14	42 ± 14
TG (mg/dl)	137 ± 86 (41.8%)^c^	125 ± 75	139 ± 88	150 ± 91^†^	126 ± 81	137 ± 84	152 ± 92^†^	125 ± 74	138 ± 88	151 ± 92^†^
FBS (mg/dl)	89 ± 25 (22.3%)^d^	90 ± 26	89 ± 23	88 ± 27	90 ± 27	89 ± 23	89 ± 27	90 ± 27	89.4 ± 23.1	88.8 ± 26.5
SBP (mmHg)	110 ± 26(28.8%)^e^	110 ± 26	110 ± 25	111 ± 27	110 ± 26	110 ± 25	111 ± 26	110 ± 26	110 ± 26	111.1 ± 26
DBP (mmHg)	73 ± 17 (28.8%)^e^	72 ± 17	73 ± 17	73 ± 18	72 ± 18	73 ± 17	73 ± 18	72 ± 18	72 ± 17	73 ± 17

From the 6th row onwards, data are presented as mean ± SD

^a^Frequency of subjects with high waist circumference: waist circumference ≥ 90 cm for both genders

^b^Frequency of subjects low HDL-C: HDL-C < 40 mg/dl in men and < 50 mg/dl in women and drug treatment

^c^Frequency of subjects high TG: TG ≥ 150 mg/dl and drug treatment

^d^Frequency of subjects high FBS: FBS ≥ 100 mg/dl and drug treatment

^e^Frequency of subjects high blood pressure: SBP ≥ 130 mmHg and DBP ≥ 85 mmHg and drug treatment

^*^Frequency differences of genotypes between MetS and without MetS, P < 0.05

^†^TT vs. CC, P < 0.05 and TT vs CT, P < 0.05

### Genotypes characteristics

Genotype frequencies of three polymorphisms followed the Hardy–Weinberg equilibrium (p > 0.01). The minor allele frequencies (MAF) were 45.8%, 46.1%, and 46.4% for the rs780094, rs780093, and rs1260326 alleles. The three SNPs are in strong LD in our population (D′ = 0.972 for rs780094 and rs780093, D′ = 0.964 for rs1260326 and rs780094, and D′ = 0.924 for rs1260326 and rs780093).

### Associations with metabolic syndrome

**Fig. 1:**
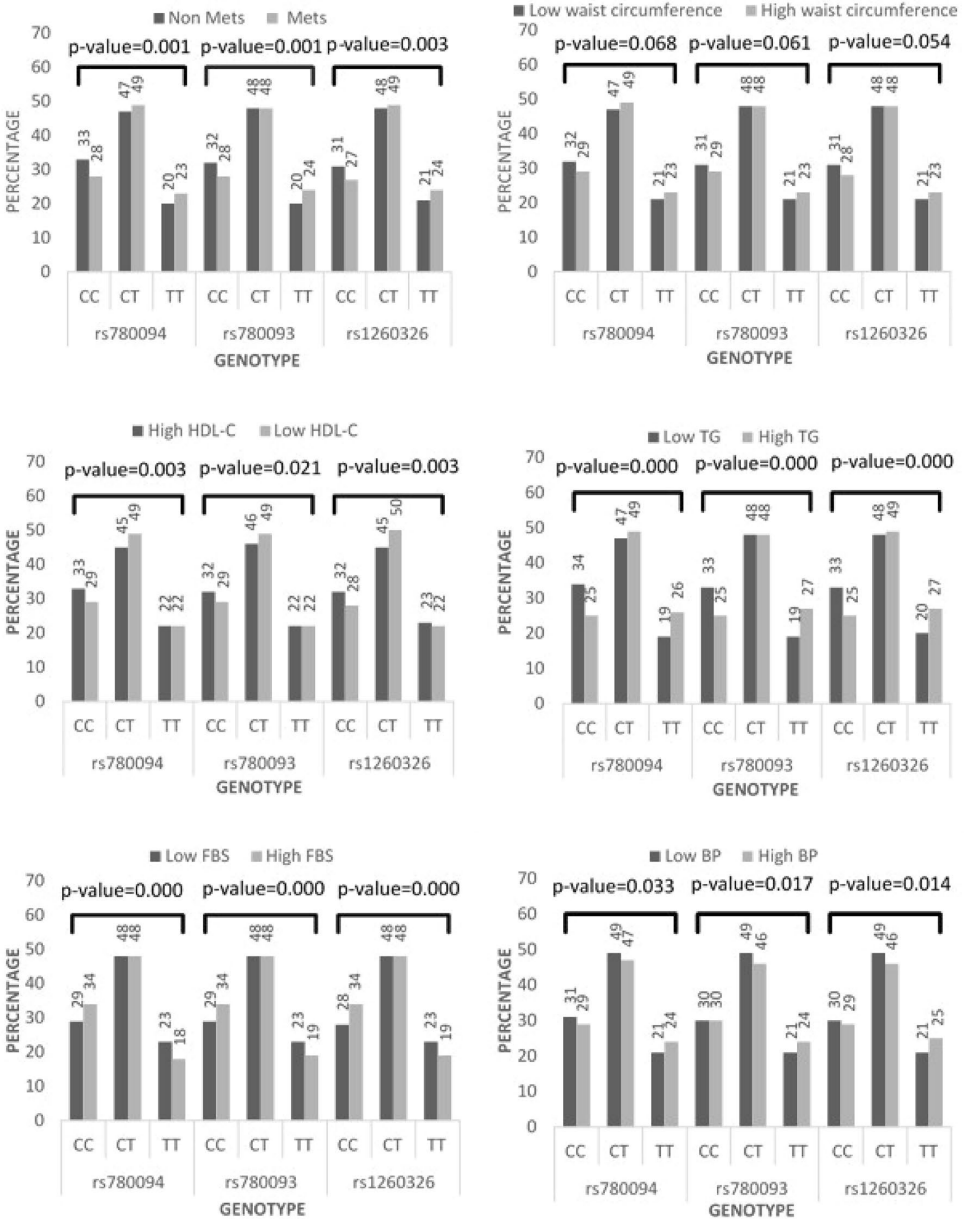
Genotypes frequencies among components of MetS groups by Chi-square test

The T alleles of the common variants of *GCKR* were frequently found in the MetS affected by non-MetS (Table 1 and Fig. 1). Results of the logistic regression analysis confirmed the association between T alleles and MetS. Adjusted odds ratios for age, gender, and smoking were 1.28 (95% CI 1.08 to 1.53), 1.34 (95% CI 1.13 to 1.6), and 1.28 (95% CI 1.08 to 1.53) (Table 2) respectively; so the homozygotes for T were at higher risk of MetS incidence.

**Table 2:** Association of GCKR SNPs with MetS and qualitative MetS components in an additive genetic model

Categorical traits	rs780094	OR (CI) model 1	Adjusted OR (CI) model 2	rs780093	OR (CI) model 1	Adjusted OR (CI) model 2	rs1260326	OR (CI) model 1	Adjusted OR (CI) model 2
Metabolic syndrome	CC (reference)	1	1	CC (reference)	1	1	CC (reference)	1	1
	CT	1.2 (1.06–1.36)*	1.22 (1.05–1.41)*	CT	1.11 (0.99–1.26)	1.14 (0.99–1.32)	CT	1.17 (1.04–1.33)*	1.19 (1.03–1.38)*
	TT	1.3 (1.12–1.51)*	1.28 (1.08–1.53)*	TT	1.32 (1.14–1.53)*	1.34 (1.13–1.6)*	TT	1.29 (1.11–1.49)*	1.28 (1.08–1.53)*
High waist circumference	CC (reference)	1	1	CC (reference)	1	1	CC (reference)	1	1
	CT	1.12 (0.99–1.27)	1.09 (0.95–1.25)	CT	1.08 (0.95–1.22)	1.08 (0.94–1.24)	CT	1.1 (0.97–1.25)	1.08 (0.94–1.24)
	TT	1.18 (1.02–1.38)*	1.13 (0.96–1.34)	TT	1.2 (1.03–1.4)*	1.17 (0.99–1.39)	TT	1.2 (1.03–1.4)*	1.17 (0.99–1.38)
Low HDL-C	CC (reference)	1	1	CC (reference)	1	1	CC (reference)	1	1
	CT	1.26 (1.1–1.44)*	1.25 (1.09–1.43)*	CT	1.21 (1.06–1.38)*	1.2 (1.04–1.37)*	CT	1.26 (1.1–1.44)*	1.25 (1.09–1.43)*
	TT	1.11 (0.94–1.3)	1.09 (0.93–1.28)	TT	1.12 (0.95–1.31)	1.1 (0.93–1.29)	TT	1.12 (0.95–1.31)	1.08 (0.91–1.27)
High TG	CC (reference)	1	1	CC (reference)	1	1	CC (reference)	1	1
	CT	1.36 (1.19–1.55)*	1.38 (1.2–1.58)*	CT	1.3 (1.14–1.48)*	1.33 (1.16–1.53)*	CT	1.36 (1.2–1.56)*	1.39 (1.21–1.6)*
	TT	1.78 (1.52–2.08)*	1.78 (1.5–2.1)*	TT	1.8 (1.54–2.1)*	1.83 (1.55–2.16)*	TT	1.84 (1.57–2.14)*	1.86 (1.57–2.19)*
High FBS	CC (reference)	1	1	CC (reference)	1	1	CC (reference)	1	1
	CT	0.85 (0.73–0.98)*	0.82 (0.71–0.96)*	CT	0.84 (0.73–0.97)*	0.83 (0.71–0.97)*	CT	0.83 (0.71–0.96)*	0.8 (0.68–0.94)*
	TT	0.68 (0.57–0.82)*	0.64 (0.52–0.77)*	TT	0.7 (0.58–0.84)*	0.65 (0.53–0.79)*	TT	0.67 (0.56–0.8)*	0.63 (0.52–0.77)*
High blood pressure	CC (reference)	1	1	CC (reference)	1	1	CC (reference)	1	1
	CT	0.99 (0.87–1.14)	0.96 (0.83–1.13)	CT	0.96 (0.83–1.1)	0.95 (0.81–1.1)	CT	0.96 (0.84–1.11)	0.93 (0.8–1.09)
	TT	1.20 (1.02–1.41)*	1.16 (0.97–1.4)	TT	1.19 (1.01–1.4)*	1.16 (0.97–1.39)	TT	1.2 (1.02–1.41)*	1.18 (0.98–1.42)

High TG: TG ≥ 150 mg/dl and drug treatment; low HDL-C: HDL-C < 40 mg/dl in men and < 50 mg/dl in women and drug treatment; high blood pressure: SBP ≥ 130 mmHg and/or DBP ≥ 85 mmHg and drug treatment; high FBS: FBS ≥ 100 mg/dl and drug treatment; high waist circumference: waist circumference ≥ 90 cm for both genders

*CI* confidence interval, *OR* odds ratio

Model 1: unadjusted

Model 2: adjusted for age, gender, and smoking

^*^P < 0.0

#### Cox regression analysis

The mean follow-up period was nearly 10 years. In Cox unadjusted model, hazard ratios were significantly higher with each additional copy of the T allele in rs780094 and rs1260326. Also, in rs780093, TT carriers had a higher incidence of MetS than CC carriers (Table 3, model 1). After adjusting for age, sex, and smoking, TT carriers of rs780094, rs780093, and rs1260326 were associated with 20%, 23%, and 21% excess risk MetS incidence, respectively (Table 3, model 2).

**Table 3:** Hazard ratios for metabolic syndrome by GCKR SNPs in an additive genetic model

Variants	Genotype	HR (CI) model 1	Adjusted HR (CI) model 2
rs780094	CC (reference)	1	1
	CT	1.14 (1.04–1.24)*	1.15 (1.05–1.26)*
	TT	1.22 (1.1–1.353)*	1.2 (1.08–1.34)*
rs780093	CC (reference)	1	1
	CT	1.08 (0.99–1.18)	1.13 (1.03–1.23)*
	TT	1.23 (1.11–1.36)*	1.23 (1.11–1.37)*
rs1260326	CC (reference)	1	1
	CT	1.11 (1.02–1.21)*	1.14 (1.04–1.25)*
	TT	1.21(1.09–1.34)*	1.21 (1.09–1.35)*

Model 1: unadjusted

Model 2: adjusted for age, gender, and smoking

*CI* confidence interval, *HR* hazard ratio

^*^P < 0.05

The values of odds ratios and hazard ratios showed that the strongest association, among the three variants, was observed between TT carriers of rs780093 and the incidence of metabolic syndrome.

### Associations with metabolic syndrome components

In baseline characteristics (first occurrence of MetS for patients with MetS), a significant difference in mean TG was observed in all genotype groups; T allele carriers of the *GCKR* variants had higher mean TG (Table 1).

Chi-square test showed that in subjects with a high TG, high blood pressure, and low HDL-C, T homozygotes of the three SNPs were more frequent. However, in people with high FBS, C homozygotes people were significantly more prevalent (Fig. 1). The first and second logic models' results determined that, with and without adjustment, C allele carriers, T allele carriers, and CT carriers in all SNPs were associated with high FBS, high TG, and low HDL-C levels, respectively. In model 1, associations were observed between high waist circumference and high blood pressure with TT carriers of the three SNPs; this association was lost after adjusting in the second model (Table 2).

The findings of the linear regression showed after age, sex, and smoking adjustment, the TT genotypes of rs780094, rs780093 and rs1260326 were associated with increased TG levels (effect per allele respectively: 0.11 mg/dl, 0.12 mg/dl and 0.12 mg/dl, p < 0.000) and reduced FBS levels (effect per allele in all SNPs: − 0.03 mg/dl, p < 0.01) (Table 4).

**Table 4:** Association of GCKR SNPs with quantitative components of MetS in an additive genetic model

Quantitative traits	rs780094			rs780093			rs1260326		
	Unstandardized coefficients^a^	Standardized coefficients^a^	P-value	Unstandardized coefficients^a^	Standardized coefficients^a^	P-value	Unstandardized coefficients^a^	Standardized coefficients^a^	P-value
	B(SE)	Β		B(SE)	β		B(SE)	β	
Waist circumference	− 0.16 (0.33)	− 0.01	0.624	− 0.01 (0.33)	0.00	0.979	0 (0.33)	0.00	0.998
HDL-C	− 0.35 (0.24)	− 0.02	0.135	− 0.34 (0.24)	− 0.02	0.15	− 0.28 (0.24)	− 0.01	0.243
FBS	− 0.95 (0.4)	− 0.03	0.017*	− 0.88 (0.4)	− 0.03	0.026*	− 0.9 (0.4)	− 0.03	0.023*
TG (natural log)	0.08 (0.01)	0.11	0.000*	0.08 (0.01)	0.12	0.000*	0.09 (0.01)	0.12	0.000*
SBP	0.07 (0.38)	0.00	0.845	0.12 (0.38)	0.00	0.758	0.12 (0.38)	0.00	0.762
DBP	0.21 (0.28)	0.01	0.465	0.3 (0.28)	0.01	0.288	0.32 (0.28)	0.01	0.251

^a^In age, gender, and smoking-adjusted linear regression model

^*^P < 0.05

## Discussion

In the present retrospective cohort study, the strongest association of MetS components with the common functional *GCKR* variants was observed between elevated TG levels and T alleles. T alleles of the variants also were associated with MetS incidence over 10 years.

We consistently replicated previous GWAS results that reported the T allele carriers of selected variants had significant associations with lower FBS and higher TG levels among MetS components [22, 23, 25, 39–42]. Among Iranian studies, two studies in children and adolescents (CASPIAN III) have previously demonstrated the association between the rs780094 risk allele and higher triglyceride levels [43, 44]. The correlation of rs1260326 and rs780094 with these MetS components has also been confirmed in our previous cross-sectional study in adults [32]. In 74 patients with non-alcoholic fatty liver disease from Tabriz city in the north-west of Iran, the lack of association between the *GCKR* rs780094 genotypes and lipid profile was observed. This finding was probably attributed to the limited sample size of this study [45].

As described above in the current study, the common variants of *GCKR* were concurrently associated with both desired and undesired MetS components and MetS, like the findings of some previous studies [22, 30]. In some other investigations, the authors suggested that the T allele's declining effect on the FBS levels has contributed to a lack of association between the *GCKR* variants and MetS [23, 46]. The obtained regression coefficient (β) values in the current study demonstrated a stronger association of the *GCKR* variants with TG than FBS. Besides, the adjusted odds ratio of the MetS components for the development of MetS (IDF criteria) in a previous study in the TLGS population, from which subjects of the present study were derived, showed that triglyceride had the highest power among other MetS components [47].

Based on our findings, CT genotypes of the *GCKR* SNPs were associated with lower HDL-C levels. In a cohort study (Atherosclerosis Risk in Communities) among white participants, the T-allele of rs780094 was associated with higher HDL-C levels. While in black participants of this study, no association was found with HDL-C [30]. This disparity in findings may be attributed more to different ethnicities than any other cause. As in another study where the participants were Taiwanese Adolescents, T-allele carriers of rs780094 were more highly represented among participants with low HDL-C levels [31].

The expression of *GCKR* produces glucokinase regulatory protein (GKRP), inhibits the enzyme GCK in competition with glucose substrate, and plays a vital role in regulating glucose storage and disposal. The GKRP itself is activated by fructose 6-phosphate binding and inactivated by fructose 1-phosphate binding. The variants investigated in this study can affect the regulation of protein by fructose 6-phosphate; for instance, based on the results of a study, GKRP from the expression of mutation in rs1260326 less regulated by fructose in the physiological range of fructose-6-phosphate concentrations, which leads to a reduction in inhibition of GCK. This reduction increases the rate of glycolysis and, therefore, glucose uptake by the liver. Enhanced glycolytic flux leads to increased triglyceride levels [35]. GKRP inhibits GCK and also stabilizes this enzyme; at low glucose concentrations. GK/GKRP complex locates in the nucleus of liver hepatocytes. As glucose levels increase, GCK enters the cytoplasm, and GKRP protects GCK against degradation by cytoplasmic proteases. Therefore, structure and function changes affect glycolysis and lipolysis regulation [30, 35, 48]. Thus defects in the mentioned pathways can lead to metabolic syndrome [49].

The minor allele frequency (MAF) of the selected GCKR SNPs was different from the other Iranian population study [43]. This difference could be Because of the current study’s greater sample size. The SNPs’ minor allele frequency in our study was similar to those in East Asia and Europe [37]. The LD pattern of 3 SNPs varies in different Hap Map populations [30]. In the TCGS population, rs780093, rs1260326, and rs780094 were in strong LD. Based on obtained β and OR, rs780093, which, based on our knowledge, had not yet been studied in the Iranian population, slightly stronger than other variants in LD, was associated with MetS incidence. The present cohort study’s strengths included using large sample size and selecting new cases of MetS, which attenuated the likelihood of any behavior changes due to awareness of metabolic abnormalities.

## Conclusions

We replicated associations of the common *GCKR* variants with metabolic syndrome components, including fasting blood sugar and triglyceride levels in Iranian adults. In the TCGS study participants, rs780094, rs780093, and rs1260326 T alleles were also associated with increased metabolic syndrome incidence. Given that in the present study, three polymorphisms were in linkage disequilibrium; therefore, whether individuals are T allele carriers, they have a risk factor for the future incidence of MetS.

## Data Availability

The datasets used and/or analyzed during the current study are available from the corresponding author on reasonable request.

## Abbreviations

CI: Confidence interval
CV: Coefficients of variation
CVDs: Cardiovascular diseases
DBP: Diastolic blood pressure
FBS: Fasting blood sugar
GCK: Glycolytic enzyme glucokinase
*GCKR*: Glucokinase regulator gene
GKRP: Glucokinase regulatory protein
GWAS: Genome-wide association studies
HDL-C: High-density lipoprotein cholesterol
HR: Hazard ratio
IDF: International Diabetes Federation
JIS: Joint Interim Statement
LN: Natural logarithm
MetS: Metabolic syndrome
OR: Odds ratios
SBP: Systolic blood pressure
SD: Standard deviation
SNPs: Single nucleotide polymorphisms
T2DM: Type 2 diabetes
TCGS: Tehran Cardiometabolic Genetics Study
TG: Triglyceride
TLGS: Tehran Lipid and Glucose Study
WC: Waist circumference
