# Correction: GCKR common functional polymorphisms are associated with metabolic syndrome and its components: a 10-year retrospective cohort study in Iranian adults

**DOI:** 10.1186/s13098-023-01079-w

**Published:** 2023-05-12

**Authors:** Asiyeh Sadat Zahedi, Mahdi Akbarzadeh, Bahareh Sedaghati-Khayat, Atefeh Seyedhamzehzadeh, Maryam S. Daneshpour

**Affiliations:** Cellular and Molecular Endocrine Research Center, Research Institute for Endocrine Sciences, Shahid Beheshti University of Medical Sciences, Tehran, POBox: 19195-4763 Iran

**Correction: Zahedi et al. Diabetol Metab Syndr _#####################_**

10.1186/s13098-021-00637-4

Following publication of the original article [1], the authors identified an error in affiliation. The correct affiliation of Maryam S. Daneshpour is given below.

Cellular and Molecular Endocrine Research Center, Research Institute for Endocrine Sciences, Shahid Beheshti University of Medical Sciences, POBox: 19,195 − 4763, Tehran, Iran

The original article has been corrected.

